# Associations of Physical Activity and Sedentary Behavior with Academic Skills – A Follow-Up Study among Primary School Children

**DOI:** 10.1371/journal.pone.0107031

**Published:** 2014-09-10

**Authors:** Eero A. Haapala, Anna-Maija Poikkeus, Katriina Kukkonen-Harjula, Tuomo Tompuri, Niina Lintu, Juuso Väistö, Paavo H. T. Leppänen, David E. Laaksonen, Virpi Lindi, Timo A. Lakka

**Affiliations:** 1 Department of Physiology, Institute of Biomedicine, University of Eastern Finland, Kuopio, Finland; 2 Department of Teacher Education, University of Jyväskylä, Jyväskylä, Finland; 3 UKK Institute for Health Promotion Research, Tampere, Finland; 4 Department of Clinical Physiology and Nuclear Medicine, Kuopio University Hospital, Kuopio, Finland; 5 Institute of Dentistry, School of Medicine, University of Eastern Finland, Kuopio, Finland; 6 Department of Psychology, University of Jyväskylä, Jyväskylä, Finland; 7 Institute of Clinical Medicine, Internal Medicine, Kuopio University Hospital, Kuopio, Finland; 8 Kuopio Research Institute of Exercise Medicine, Kuopio, Finland; University of Bath, United Kingdom

## Abstract

**Background:**

There are no prospective studies that would have compared the relationships of different types of physical activity (PA) and sedentary behavior (SB) with academic skills among children. We therefore investigated the associations of different types of PA and SB with reading and arithmetic skills in a follow-up study among children.

**Methods:**

The participants were 186 children (107 boys, 79 girls, 6–8 yr) who were followed-up in Grades 1–3. PA and SB were assessed using a questionnaire in Grade 1. Reading fluency, reading comprehension and arithmetic skills were assessed using standardized tests at the end of Grades 1–3.

**Results:**

Among all children more recess PA and more time spent in SB related to academic skills were associated with a better reading fluency across Grades 1–3. In boys, higher levels of total PA, physically active school transportation and more time spent in SB related to academic skills were associated with a better reading fluency across the Grades 1–3. Among girls, higher levels of total PA were related to worse arithmetic skills across Grades 1–3. Moreover, total PA was directly associated with reading fluency and arithmetic skills in Grades 1–3 among girls whose parents had a university degree, whereas these relationships were inverse in girls of less educated parents.

**Conclusions:**

Total PA, physically active school transportation and SB related to academic skills may be beneficial for the development of reading skills in boys, whereas factors that are independent of PA or SB may be more important for academic skills in girls.

**Trial Registration:**

ClinicalTrials.gov: NCT01803776

## Introduction

Physical activity (PA), particularly physically active transportation, is decreasing, whereas sedentary behaviors, especially watching TV, sitting at the computer and playing video games, are increasing among children in developed countries [1]–[4]. This trend is a major public health problem because sedentary lifestyle in childhood has been found to increase the risk of chronic diseases in adulthood [5], [6].

The results of some cross-sectional studies suggest that lower levels of PA is associated with a poorer academic achievement among children [7]–[10]. Moreover, intervention studies have provided evidence that implementing 90 minutes of moderate-to-vigorous PA per week within a school day [11], adding 60 minutes of physical education per day [12] or increasing after-school PA for 40 minutes per day [13] improves academic achievement among children. However, some intervention and cross-sectional studies have reported only a weak or non-significant relationship between PA and academic achievement in children [14]–[18].

One explanation for the inconsistent results of previous studies may be that various types of PA and sedentary behavior are differently related to academic achievement among children and adolescents. Some prior studies have found direct associations of physical education and extracurricular PA with academic achievement in children [19]. The results of one study suggested that engaging in sports is more strongly associated with academic achievement than total PA in adolescents [20]. Moreover, one 20-minute bout of moderate-intensity PA has been shown to acutely improve performance in an academic achievement test in children [21], [22]. However, there is limited evidence on the associations of other types of PA, such as physically active school transportation or recess PA with measures of academic achievement such as grades, standardized test scores or reading and arithmetic skills.

Several studies have shown that acute and long-term PA improves cognitive functions, such as attention, concentration and working memory, which underlie academic achievement [23]–[25]. Kamijo and co-workers [26] and Chaddock-Heyman and colleagues [27] showed an improved working memory and cognitive control, respectively, after a 9-month PA intervention in 8–9-year-old children. Moreover, Davis and associates reported enhanced executive functions after a 13-week PA intervention among overweight children [13]. There is also some evidence that PA during recess improves attention, concentration and on-task behavior in children [19], [28]. In another study more time spent walking or bicycling to and from school was related to better cognitive functions independent of total PA in adolescents [29].

Most studies on the associations of different types sedentary behavior with academic achievement have concentrated on screen-based sedentary behaviors such as watching TV and playing with the computer [30]. Whereas one study found an inverse association between TV watching and academic achievement in children and adolescents [30], another study suggested that a longer time spent watching TV was related to a better academic achievement in children [31]. Screen time was inversely related to grade-point average but total sedentary time was not associated with academic achievement in Finnish children [10]. Having a TV set in the bedroom has also been associated with a poorer academic achievement [31]. Access to a home computer, however, has been associated with an improved academic achievement in 8-year-old children [31].

Only a few cross-sectional studies have compared different types of PA and sedentary behavior in relation to measures of academic achievement such as reading and arithmetic skills among children and adolescents. Moreover, there are no follow-up studies on these topics in these age groups. We therefore investigated the associations of different types of PA and sedentary behavior in Grade 1 with reading and arithmetic skills in Grades 1–3 and the differences in academic skills in Grades 1–3 between children who were in the upper and lower halves of PA and sedentary behavior in Grade 1 among a population sample of Finnish primary school children. We also studied whether sex and parental education modified the associations of PA and sedentary behavior with academic skills.

## Methods

### Study design and study population

Data for the present analyses were derived from the Physical Activity and Nutrition in Children (PANIC) Study [32] and the First Steps Study [33] that are two independent studies that are being conducted simultaneously among primary school children in the City of Kuopio, Finland. The PANIC Study is a controlled exercise and diet intervention study in a population sample of primary school children. Altogether 736 children 6–8 years of age who were in Grade 1 in 2007–2009 were invited to participate in the baseline examinations in 2007–2009. Of the 736 invited children, 512 (70%) participated. The First Steps Study is a 5-year follow-up study conducted in 2006–2011 in a population-based sample of 2000 children from four municipalities in different parts of Finland. Altogether 207 children from the City of Kuopio participated in both the PANIC Study and the First Steps Study. Data on PA and sedentary behavior in Grade 1 were obtained from the PANIC Study and reading and arithmetic skills at the end of Grades 1, 2 and 3 from the First Steps Study. Complete data on PA, sedentary behaviors, reading and arithmetic skills, as well as other variables used in the present analyses were available for 186 children (107 boys, 79 girls).

Boys in the current study sample had more likely entered puberty than other boys in the PANIC Study sample (*P* = 0.012). Girls in the current study sample had lower levels of total PA (*P* = 0.015) and unsupervised PA (*P* = 0.002), spent less time in sedentary behavior related to academic skills (*P* = 0.002) and spent more time using computer and playing video games (*P* = 0.031) and sitting and lying for a rest (*P* = 0.032) than other girls in the PANIC Study sample. There were no differences in academic skills between the current study sample and other children in the First Steps Study sample in boys or girls.

The PANIC Study protocol was approved by the Research Ethics Committee of the Hospital District of Northern Savo, Kuopio. The First Steps Study protocol was approved by the Research Ethics Committee of the University of Jyväskylä. All participating children and their parents provided a written informed consent.

### Assessment of physical activity and sedentary behavior

PA and sedentary behavior in Grade 1 were assessed by the PANIC Physical Activity Questionnaire administered by the children together with their parents [34].

The types of PA included *organized sports, organized exercise other than sports, unsupervised PA, physically active school transportation (such as walking and bicycling)* and *PA during recess*. In Finnish schools, 1.5 hours of physical education per week are compulsory for all children in Grades 1–9. We did not include physical education in analyses concerning the different types of PA, because time spent in physical education was the same for all children. We did, however, include time spent in physical education in total PA. The frequency of each type of PA and the average duration of the sessions of the PA were asked. The amount of PA was calculated by multiplying the frequency of the PA with the average duration of the PA session and was expressed in minutes per day. Total PA was calculated by summing-up the amounts of different types of PA and was expressed in minutes per day.

The types of sedentary behavior during leisure time included screen-based sedentary behavior (*watching TV and videos*, *using the computer* and *playing video games*, *using a mobile phone and playing mobile games*), sedentary behavior related to music (*listening to music*, *playing music*), sedentary behavior related to academic skills (*reading*, *writing*), sedentary behavior related to arts, crafts and games (*drawing*, *doing arts and crafts*, *playing board and card games*) and *sitting and lying for a rest*. Times spent in each sedentary behavior separately on weekdays and weekends were asked and were expressed in minutes per day. The amount of total sedentary behavior was calculated by summing-up the times spent in each sedentary behavior and was expressed in minutes per day weighted by the number of weekdays and weekend days.

We validated the PANIC Physical Activity Questionnaire using the Actiheart monitor (Actiheart, CamNtech, Cambridge, UK) combining heart rate and accelerometry measurements in a subsample of 38 children examined at baseline of the PANIC Study [34]. Total PA measured by the questionnaire correlated positively with total PA measured by the Actiheart monitor (r = 0.37, *P* = 0.033).

### Assessment of academic skills

Reading fluency was assessed using a group-administered speeded subtest of the nationally normed reading achievement test battery (ALLU) [35]. The test score was the number of correct answers, ranging from 0 to 80, during a 2-minute time limit for items that involved identifying the correct word from four phonologically similar alternatives linked to an adjoining picture.

Reading comprehension was assessed with a group-administered subtest from the ALLU test battery [35]. After reading a short text, children were asked to answer to 12 multiple-choice questions including facts, causal relationships, interpretations or conclusions drawn from the text. The test score was the number of correct answers, ranging from 0 to 12, during the 30-minute test period when children were allowed to refer to the original text.

Arithmetic skills were assessed using a basic arithmetic test [36] with a set of visually presented addition and subtraction tasks. Children were asked to perform as many calculations as they could. The test score was the number of correct answers, ranging from 0 to 28, during the 3-minute time limit.

### Assessment of confounding factors

Body fat percentage and lean body mass were measured after emptying the bladder, in a supine position, in light clothing and after removing all metal objects by a Lunar dual energy X-ray absorptiometry (DXA) device (Lunar Prodigy Advance; GE Medical Systems, Madison, WI, USA) [37]. Cardiovascular performance was assessed using a maximal exercise test with an electromagnetically-braked Ergoline cycle ergometer (Ergoselect 200 K; Ergoline, Bitz, Germany) and was defined as maximal workload in Watts per kg of lean body mass [38], [39]. Overall motor performance was calculated as the sum of Z-scores for the 50-meter shuttle run test time (inverse), errors in the flamingo balance test (inverse) and the number of cubes moved in the box and block test [40].

The risk of reading disability was assessed using children’s scores in the kindergarten-age tests of letter knowledge, phonemic awareness and rapid automatized naming, and family risk of reading difficulties defined positive if the mother or father reported in a questionnaire that she or he had had “mild” or “severe” problems in reading at school age [41]. The children were defined as being at risk of reading disability if their score was at or below the 15th percentile (about one standard deviation below the mean of the sample of approximately 2000 children) in at least two of the three skills or in one skill in the presence of family risk.

The parents were asked to report their completed or ongoing educational degrees (vocational school or less, polytechnic or university). The degree of the more educated parent was used in the analyses. The research physician performed a standard clinical examination, including the assessment of pubertal status. The boys were classified as having entered clinical puberty if their testicular volume assessed by orchidometer was >3 ml, and the girls were defined as having entered puberty if their breast development in scales described by Tanner was >B1 [42]. Because only a few children had entered clinical puberty, we also used the current height as a percentage of predicted adult height as a measure of maturity, as explained in detail earlier [40].

### Statistical methods

We performed statistical analyses using the SPSS software, Version 21 (IBM, Armonk, NY, USA). We used the Student’s t-test, the Mann-Whitney U test and the Chi-square test to compare background characteristics, PA and sedentary behavior between the boys and the girls.

We examined whether different types of PA and sedentary behavior during Grade 1 as continuous variables were independently associated with academic skills during Grades 1, 2 and 3 using multivariate linear regression analyses. In the first step, we forced age, sex, parental education and the PANIC study group (intervention vs. control) as basic covariates, and entered different types of PA and sedentary behavior one by one into the models. If the associations of different types of PA and sedentary behavior with academic skills were statistically significant after adjustment for the basic covariates, we also forced clinical puberty, the current height as a percentage of predicted adult height, body fat percentage, cardiovascular performance, motor performance, the risk of reading disability or the different types of PA or sedentary behavior one by one as additional covariates into the models. Variables that were strongly correlated were not forced into the same models to avoid collinearity problem. We repeated the linear regression analyses in the boys and the girls separately after adjustment for the same basic and additional covariates, except sex.

We used analyses of covariance (ANCOVA) with repeated measures to investigate whether there were differences in academic skills in Grades 1–3 between children who were in the lower and upper halves of different types of PA or sedentary behavior in Grade 1 using sex-specific medians as cut-offs. However, we categorized the children as those who participated in organized sports and those who did not, because a relatively small number of children participated in organized sports. The ANCOVA data were adjusted using the same principle as the linear regression analysis data.

The selection of covariates for the multivariate linear regression analyses and the ANCOVAs was based on the previous and current evidence on their associations with PA, sedentary behavior and academic skills. The PANIC study group (intervention vs. control) was used as a covariate, because the intervention in the PANIC Study started before the assessments of academic skills in the First Steps Study. Because sex modified some associations of PA and sedentary behavior with academic skills, we also conducted the multivariate linear regression analyses and the ANCOVAs in the boys and the girls. We estimated statistical power for our analyses using the G*Power software [43], [44]. One hundred and ninety one observations was needed to observe the correlation of 0.2 at the power of 0.80 when statistical significance level was set at *P*<0.05. Moreover, a correlation coefficient needed to reveal statistical significance at the level of *P*<0.05 was 0.26 in boys and 0.30 in girls.

## Results

### Basic characteristics

The boys were slightly older and physically more active and spent more time on the computer and playing video games, less time in sedentary behavior related to music and less time in sedentary behavior related to arts, crafts and games than the girls (Table 1). The boys also had reached a lower proportion of their predicted adult height, had a lower body fat percentage and a higher maximal workload per lean body mass in the exercise test and were more likely to be at risk of reading disability than the girls. The girls were better in reading comprehension in Grade 2 and in reading fluency in Grade 3 than the boys.

**Table 1:** Basic characteristics of 186 children (107 boys and 79 girls).

	All	Boys	Girls	*P*-value
Age (years)	7.7 (0.4)	7.7 (0.4)	7.6 (0.3)	0.033
Parental education (%)				0.095
Vocational school or less	20.0	23.6	15.2	
Polytechnic	39.5	33.0	48.1	
University	40.5	43.4	36.7	
PANIC Study group (%)				0.907
Intervention	68.8	69.2	68.4	
Control	31.2	30.8	31.6	
Pubertal status (%)				0.894
Prepubertal	95.1	95.2	94.8	
Pubertal	4.9	4.8	5.2	
The current height as a percentage of predicted adult height	74.6 (3.6)	72.4 (2.6)	77.2 (2.7)	<0.001
Body fat percentage	18.4 (11.8)	14.9 (11.2)	20.9 (13.7)	<0.001
Maximal workload per lean body mass	3.6 (0.5)	3.7 (0.5)	3.4 (0.4)	<0.001
Motor performance score	0.09 (2.0)	0.08 (2.0)	0.10 (2.0)	0.985
Risk for reading disability (%)	13.6	17.9	7.7	0.037
Total physical activity (min/d)	105 (39.9)	112 (43.5)	95.4 (32.2)	0.010
Supervised exercise (min/d)	13.6 (12.7)	14.3 (12.9)	12.6 (12.6)	0.207
Organized sports (min/d)	8.4 (11.5)	8.6 (11.4)	8.0 (11.7)	0.503
Organized exercise other than sports (min/d)	5.2 (5.9)	5.7 (6.3)	4.6 (5.4)	0.240
Unsupervised physical activity (min/d)	44.2 (30.6)	51.1 (32.0)	35.0 (26.1)	<0.001
Physically active school transportation (min/d)	18.7 (15.1)	17.6 (15.7)	20.2 (14.1)	0.054
Physical activity during recess (min/d)	22.0 (6.1)	23.0 (6.0)	20.8 (5.9)	0.013
Total sedentary behavior (min/d)	215 (110)	206 (102)	227 (119)	0.247
Screen-based sedentary behavior (min/d)	106 (54.4)	111 (60.0)	99.5 (45.3)	0.110
Watching TV and videos (min/d)	68.9 (32.6)	66.0 (31.9)	74.1 (32.9)	0.146
Using computer and playing video games (min/d)	34.2 (33.8)	42.0 (37.4)	23.5 (24.5)	<0.001
Using mobile phone and playing mobile games (min/d)	3.3 (8.4)	4.3 (11.3)	1.9 (5.7)	0.056
Sedentary behavior related to academic skills (min/d)	21.4 (38.6)	22.9 (38.6)	21.4 (36.4)	0.657
Sedentary behavior related to music (min/d)	16.0 (23.0)	11.9 (16.3)	21.6 (28.9)	0.026
Sedentary behavior related to arts, crafts and games (min/d)	46.1 (47.7)	38.6 (45.7)	60.0 (42.9)	<0.001
Sitting and lying for a rest (min/d)	11.9 (26.9)	11.7 (28.0)	12.2 (25.7)	0.836
Academic skills in Grade 1				
Reading fluency	18.8 (9.2)	18.2 (9.2)	19.6 (8.1)	0.128
Reading comprehension	4.9 (3.3)	4.8 (3.4)	5.2 (3.2)	0.343
Arithmetic skills	10.4 (4.2)	10.6 (4.3)	10.1 (3.9)	0.495
Academic skills in Grade 2				
Reading fluency	25.2 (8.2)	24.9 (9.1)	25.6 (6.9)	0.297
Reading comprehension	7.8 (3.0)	7.2 (3.2)	8.5 (2.6)	0.007
Arithmetic skills	15.6 (5.2)	15.4 (5.6)	15.8 (4.5)	0.827
Academic skills in Grade 3				
Reading fluency	36.6 (8.7)	35.5 (8.9)	38.1 (8.3)	0.040
Reading comprehension	8.9 (2.2)	8.6 (2.4)	9.2 (1.7)	0.226
Arithmetic skills	19.7 (4.6)	19.8 (4.5)	19.7 (4.8)	0.854

Basic characteristics are displayed as means (SD), medians (IQRs) or percentages (%). Differences in basic characteristics between girls and boys were analyzed with the Student’s t-test or Mann-Whitney U test for continuous variables and chi-square test for categorical variables.

### Physical activity and academic skills among all children

Among all children, PA during recess in Grade 1 as a continuous variable was directly associated with reading fluency in Grades 1–2 after adjustment for age, sex, parental education and the PANIC study group (Table 2). Children who were in the upper half of PA during recess in Grade 1 (≥median of 20 min/d) had a better reading fluency in Grades 1–3 than those who were in the lower half after these adjustments (*P* = 0.015, partial eta-squared, η^2p^ = 0.032). Further adjustment for clinical puberty, the current height as a percentage of predicted adult height, body fat percentage, cardiovascular performance, motor performance, the risk of reading disability or the different types of sedentary behavior had no effect on these associations or differences (data not shown).

**Table 2:** The associations of physical activity in Grade 1 with academic skills in Grades 1–3 among 186 children (107 boys and 79 girls).

	Reading fluency			Reading comprehension			Arithmetic skills		
	All	Boys	Girls	All	Boys	Girls	All	Boys	Girls
	Grade 1			Grade 1			Grade 1		
Total physical activity (min/d)	0.11	**0.23** *	−0.17	0.06	0.16	−0.12	−0.00	0.08	−**0.25** *
Supervised exercise (min/d)	0.08	0.16	−0.08	0.06	0.08	0.01	0.08	0.14	−0.05
Organized sports (min/d)	0.05	0.09	−0.03	0.00	0.04	−0.08	0.07	0.09	0.03
Organized exercise other than sports (min/d)	0.09	0.18	−0.13	0.13	0.09	0.18	0.04	0.14	−0.17
Unsupervised physical activity (min/d)	0.02	0.10	−0.14	−0.03	0.05	−0.11	−0.03	0.04	−0.19
Physically active school transportation (min/d)	0.12	0.26**	−0.13	0.14	0.25**	−0.08	−0.07	0.01	−0.21
Physical activity during recess (min/d)	0.19**	0.14	0.22	0.03	0.02	0.06	0.14	0.12	0.13
	**Grade 2**			**Grade 2**			**Grade 2**		
Total physical activity (min/d)	0.08	**0.22** *	−**0.28** *	0.07	0.12	−0.02	0.05	0.15	−0.17
Supervised exercise (min/d)	0.04	0.10	−0.09	0.06	0.07	−0.04	0.10	0.17	−0.04
Organized sports (min/d)	−0.02	0.04	−0.08	0.02	0.05	−0.10	0.11	0.12	0.06
Organized exercise other than sports (min/d)	0.09	0.14	−0.05	0.09	0.06	0.13	0.02	0.15	−0.22
Unsupervised physical activity (min/d)	−0.00	0.11	−**0.23** *	0.01	0.03	0.07	−0.01	0.05	−0.12
Physically active school transportation (min/d)	0.13	0.26**	−0.16	0.06	0.16	−0.21	0.04	0.12	−0.14
Physical activity during recess (min/d)	**0.17** *	0.18	0.13	0.13	0.14	0.18	0.11	0.14	0.09
	**Grade 3**			**Grade 3**			**Grade 3**		
Total physical activity (min/d)	0.06	0.14	−0.12	−0.03	0.05	−0.13	−0.03	0.04	−0.20
Supervised exercise (min/d)	0.04	0.05	0.01	−0.00	−0.01	0.03	0.04	0.03	0.03
Organized sports (min/d)	0.02	−0.01	0.05	−0.01	−0.04	0.03	0.07	−0.03	0.17
Organized exercise other than sports (min/d)	0.04	0.13	−0.10	0.01	0.04	−0.00	−0.04	0.13	−0.29**
Unsupervised physical activity (min/d)	0.01	0.04	−0.06	−0.06	−0.03	−0.06	−0.07	−0.01	−0.18
Physically active school transportation (min/d)	0.08	**0.22** *	−0.19	0.03	0.15	−**0.24** *	−0.03	0.09	−0.20
Physical activity during recess (min/d)	0.14	0.11	0.10	0.09	0.15	0.09	0.12	0.07	0.22

Results of linear regression analyses. Results are displayed as standardized regression coefficients. Background variables including age, sex, parental education and the PANIC study group were entered in a linear regression model in the Step 1 and all other variables were entered separately into the model in the Step 2.

*P<0.05, **P<0.01, †P<0.001.

Organized sports in Grade 1 as a continuous variable was not associated with academic skills in Grades 1–3 after adjustment for age, sex, parental education and the PANIC study group (Table 2). However, children who participated in any organized sports in Grade 1 had better arithmetic skills in Grades 1–3 than those who did not engage in organized sports after these adjustments (*P* = 0.015, η^2p^ = 0.032). Further adjustment for clinical puberty, the current height as a percentage of predicted adult height, body fat percentage, cardiovascular performance, motor performance, the risk of reading disability or the different types of sedentary behavior had no effect on these differences (data not shown).

Physically active school transportation in Grade 1 as a continuous variable was not associated with academic skills in Grades 1–3 after adjustment for age, sex, parental education and the PANIC study group (Table 2). However, children who were in the upper half of physically active school transportation in Grade 1 (≥median of 14 min/d) had a better reading fluency in Grades 1–3 than those who were in the lower half after these adjustments (*P* = 0.038, η^2p^ = 0.024). Further adjustment for clinical puberty, the current height as a percentage of predicted adult height, body fat percentage, cardiovascular performance, motor performance, the risk of reading disability or the different types of sedentary behavior had no effect on these differences (data not shown).

### Physical activity and academic skills among boys

**Figure 1:**
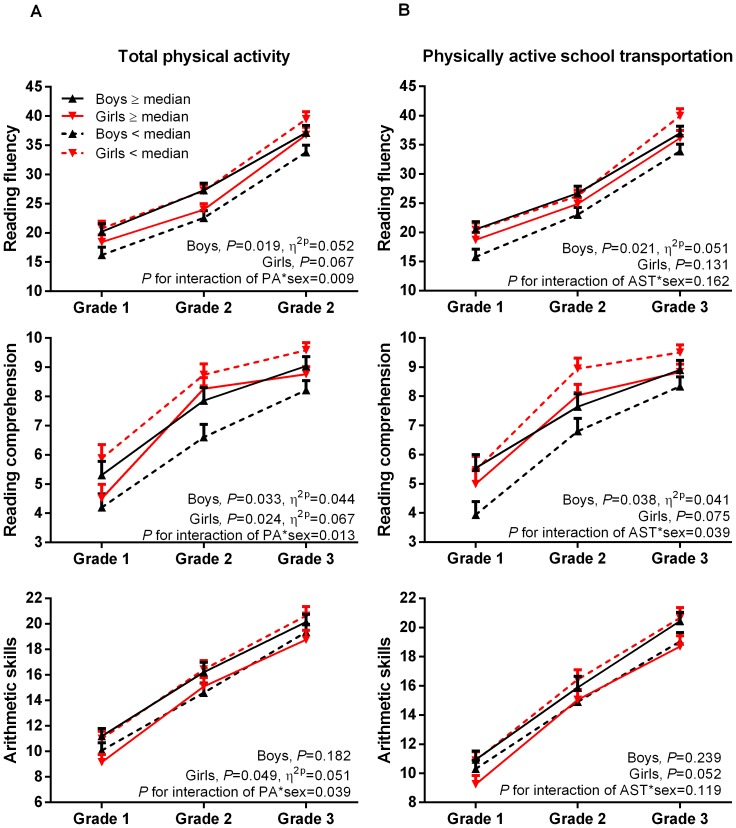
The differences in academic skills in Grades 1–3 between children who were in the upper and lower halves of total physical activity (A) and physically active school transportation (B) in Grade 1 among 107 boys and 79 girls. The data were adjusted for age, parental education, the PANIC study group (intervention vs. control) from the analyses of covariance with repeated measures. Error bars represent standard errors of the mean (SEM).

Total PA in Grade 1 as a continuous variable was directly associated with reading fluency in Grades 1–2 after adjustment for age, parental education and the PANIC study group (Table 2). The relationship between total PA in Grade 1 and reading fluency in Grade 2 was weakened after adjustment for body fat percentage (β = 0.19, *P* = 0.059), cardiovascular performance (β = 0.19, *P* = 0.072) or motor performance (β = 0.19, *P* = 0.049). Boys who were in the upper half of total PA in Grade 1 (≥median of 110 min/d) had a better reading fluency and reading comprehension in Grades 1–3 than boys who were in the lower half after adjustment for age, parental education and the PANIC study group (Figure 1A). These differences were no longer statistically significant after further adjustment for body fat percentage, cardiovascular performance or motor performance (data not shown). Further adjustment for clinical puberty, the current height as a percentage of predicted adult height, the risk of reading disability or the different types of sedentary behavior had no effect on these associations or differences (data not shown).

Physically active school transportation in Grade 1 as a continuous variable was directly related to reading fluency in Grades 1–3 and reading comprehension in Grade 1 after adjustment for age, parental education and the PANIC study group (Table 2). Further adjustment for clinical puberty, the current height as a percentage of predicted adult height, body fat percentage, cardiovascular performance, motor performance, the risk of reading disability or the different types of sedentary behavior had no effect on these associations (data not shown). Boys who were in the upper half of physically active school transportation in Grade 1 (≥median of 14 min/d) had a better reading fluency and reading comprehension in Grades 1–3 than boys who were in the lower half after adjustment for age, parental education and the PANIC study group (Figure 1B). These differences were no longer statistically significant after further adjustment for motor performance (data not shown). Other adjustments had no effect on these differences (data not shown).

### Physical activity and academic skills among girls

Total PA in Grade 1 as a continuous variable was inversely associated with reading fluency in Grade 2 and arithmetic skills in Grade 1 after adjustment for age, parental education and the PANIC study group (Table 2). Further adjustment for body fat percentage weakened the association between total PA in Grade 1 and arithmetic skills in Grade 1 (β = −0.19, *P* = 0.096). Other adjustments had no effect on these relationships (data not shown). Girls who were in the upper half of total PA in Grade 1 (≥median of 97 min/d) had a worse reading comprehension and worse arithmetic skills in Grades 1–3 than girls who were in the lower half after adjustment for age, parental education and the PANIC study group (Figure 1A). These differences were no longer statistically significant after additional adjustment for body fat percentage or motor performance (data not shown). Other adjustments had no effect on these differences (data not shown). Girls in the lower half of total PA in Grade 1 had a better reading fluency in Grades 1–3 than girls in the upper half among girls whose parents had no university degree, but girls in the upper half had a better reading fluency than girls in the lower half of total PA among girls whose parents had a university degree (*P* = 0.009 for interaction). Parental education similarly modified the associations of total PA with arithmetic skills in Grades 1–3 in the girls (*P* = 0.002 for interaction).

Unsupervised PA in Grade 1 as a continuous variable was inversely related to reading fluency in Grade 2 after adjustment for age, parental education and the PANIC study group (Table 2). This association was weakened after further adjustment for body fat percentage (β = −0.20, *P* = 0.077). Other adjustments had no effect on this association.

Organized exercise other than sports in Grade 1 as a continuous variable was inversely related to arithmetic skills in Grade 3 after adjustment for age, parental education and the PANIC study group (Table 2). Physically active school transportation in Grade 1 was inversely associated with reading comprehension in Grade 3 after these adjustments (Table 2). Further adjustments had no effect on these associations (data not shown).

### Sedentary behavior and academic skills among all children

Sedentary behavior related to academic skills as a continuous variable was directly related to reading fluency in Grades 1–3 after adjustment for age, sex, parental education and the PANIC study group (Table 3). Children who were in the upper half of sedentary behavior related to academic skills in Grade 1 (≥median of 23 min/d in boys and 21 min/d in girls) had a better reading fluency in Grades 1–3 than children who were in the lower half (*P*<0.001, η^2p^ = 0.068). Further adjustment for clinical puberty, the current height as a percentage of predicted adult height, body fat percentage, cardiovascular performance, motor performance, the risk of reading disability or the different types of PA had no effect on these associations or differences (data not shown).

**Table 3:** The associations of sedentary behaviors in Grade 1 with academic skills in Grades 1–3 among 186 children (107 boys and 79 girls).

	Reading fluency			Reading comprehension			Arithmetic skills		
	All	Boys	Girls	All	Boys	Girls	All	Boys	Girls
	Grade 1			Grade 1			Grade 1		
Total SB (min/d)	0.13	0.27**	−0.06	0.05	0.16	−0.09	0.05	0.18	−0.11
Screen-based SB (min/d)	0.05	0.08	−0.01	−0.03	−0.01	−0.10	0.13	0.14	0.10
Watching television and videos (min/d)	0.04	0.06	0.01	−0.05	−0.04	−0.08	0.10	0.11	0.09
Using computer and playing video games (min/d)	0.01	0.03	−0.05	−0.01	0.00	−0.06	0.08	0.08	0.08
Using mobile phone and playing mobile games (min/d)	0.13	0.15	0.04	0.02	0.05	−0.12	0.10	0.14	−0.05
Sedentary behavior related to academic skills (min/d)	**0.25†**	**0.40†**	0.07	0.14	**0.22** *	0.05	0.08	0.17	−0.01
Sedentary behavior related to music (min/d)	−0.03	0.04	−0.08	−0.02	0.02	−0.05	−0.09	0.06	−0.22
Sedentary behavior related to, arts, crafts and games (min/d)	0.02	0.11	−0.11	−0.02	0.08	−0.12	−0.09	−0.00	−0.19
Sitting and lying for a rest (min/d)	0.09	0.15	−0.03	0.13	**0.21** *	−0.00	0.05	0.12	−0.06
	**Grade 2**			**Grade 2**			**Grade 2**		
Total SB (min/d)	0.11	0.18	0.02	−0.07	−0.02	−0.14	−0.01	0.15	−**0.23** *
Screen-based SB (min/d)	0.05	0.00	0.12	−0.06	−0.06	−0.13	0.08	0.15	−0.05
Watching television and videos (min/d)	0.06	0.03	0.09	−0.08	−0.09	−0.09	0.07	0.18	−0.11
Using computer and playing video games (min/d)	0.02	−0.02	0.12	−0.04	−0.04	−0.13	0.06	0.09	0.03
Using mobile phone and playing mobile games (min/d)	−0.01	−0.01	−0.07	0.07	0.07	0.03	0.01	−0.03	0.07
Sedentary behavior related to academic skills (min/d)	**0.19** *	0.30**	0.04	0.08	0.17	−0.03	0.01	0.13	−0.16
Sedentary behavior related to music (min/d)	−0.11	−0.04	−0.18	−0.06	0.05	−0.14	−0.10	0.09	−**0.28** *
Sedentary behavior related to, arts, crafts and games (min/d)	0.07	0.15	−0.03	−0.14	−0.10	−0.20	−0.08	0.02	−0.20
Sitting and lying for a rest (min/d)	0.11	0.12	0.08	0.00	0.04	−0.07	−0.01	0.02	−0.05
	**Grade 3**			**Grade 3**			**Grade 3**		
Total SB (min/d)	0.08	**0.24** *	−0.10	0.06	0.11	0.03	−0.07	0.12	−**0.25** *
Screen-based SB (min/d)	0.05	0.08	0.04	0.05	0.03	0.10	0.03	0.08	−0.06
Watching television and videos (min/d)	0.05	0.11	−0.02	0.09	0.06	0.14	0.03	0.10	−0.07
Using computer and playing video games (min/d)	0.01	−0.01	0.09	−0.01	0.00	−0.02	0.02	0.04	−0.03
Using mobile phone and playing mobile games (min/d)	0.08	0.08	0.09	0.04	0.02	0.11	0.02	−0.02	0.02
Sedentary behavior related to academic skills	0.22**	**0.38†**	0.04	0.08	0.12	0.06	0.02	0.19	−0.14
Sedentary behavior related to music (min/d)	−0.12	−0.03	−0.19	−0.13	−0.13	−0.15	−**0.15** *	−0.01	−**0.23** *
Sedentary behavior related to, arts, crafts and games (min/d)	0.00	0.14	−0.16	0.06	0.14	−0.04	−**0.15** *	0.02	−0.29**
Sitting and lying for a rest (min/d)	0.04	0.12	−0.09	0.09	0.07	0.15	0.01	0.06	−0.06

Results of linear regression analyses. Results are displayed as standardized regression coefficients. Background variables including age, sex, parental education and the PANIC study group were entered in a linear regression model in the Step 1 and all other variables were entered separately into the model in the Step 2. ^1^Time computed as a sum of time spent listening and playing music, doing hands and crafts, drawing and playing board and card games. SB = sedentary behavior.

*P<0.05, **P<0.01, †P<0.001.

Sedentary behavior related to music and sedentary behavior related to arts, crafts and games as continuous variables were inversely associated with arithmetic skills in Grade 3 after adjustment for age, sex, parental education and the PANIC Study group (Table 3). The association of sedentary behavior related to music with arithmetic skills in Grade 3 was no longer statistically significant after further adjustment for motor performance (data not shown). The association of sedentary behavior related to arts, crafts and games with arithmetic skills in Grade 3 was no longer statistically significant after additional adjustment for cardiovascular performance (data not shown). Other adjustments had no effect on these associations (data not shown).

### Sedentary behavior and academic skills among boys

Total sedentary behavior in Grade 1 was directly associated with reading fluency in Grades 1 and 3 after adjustment for age, parental education and the PANIC study group (Table 3). However, these relationships were no longer statistically significant after further adjustment for sedentary behavior related to academic skills (data not shown). Further adjustments had no effect on these associations or differences (data not shown).

**Figure 2:**
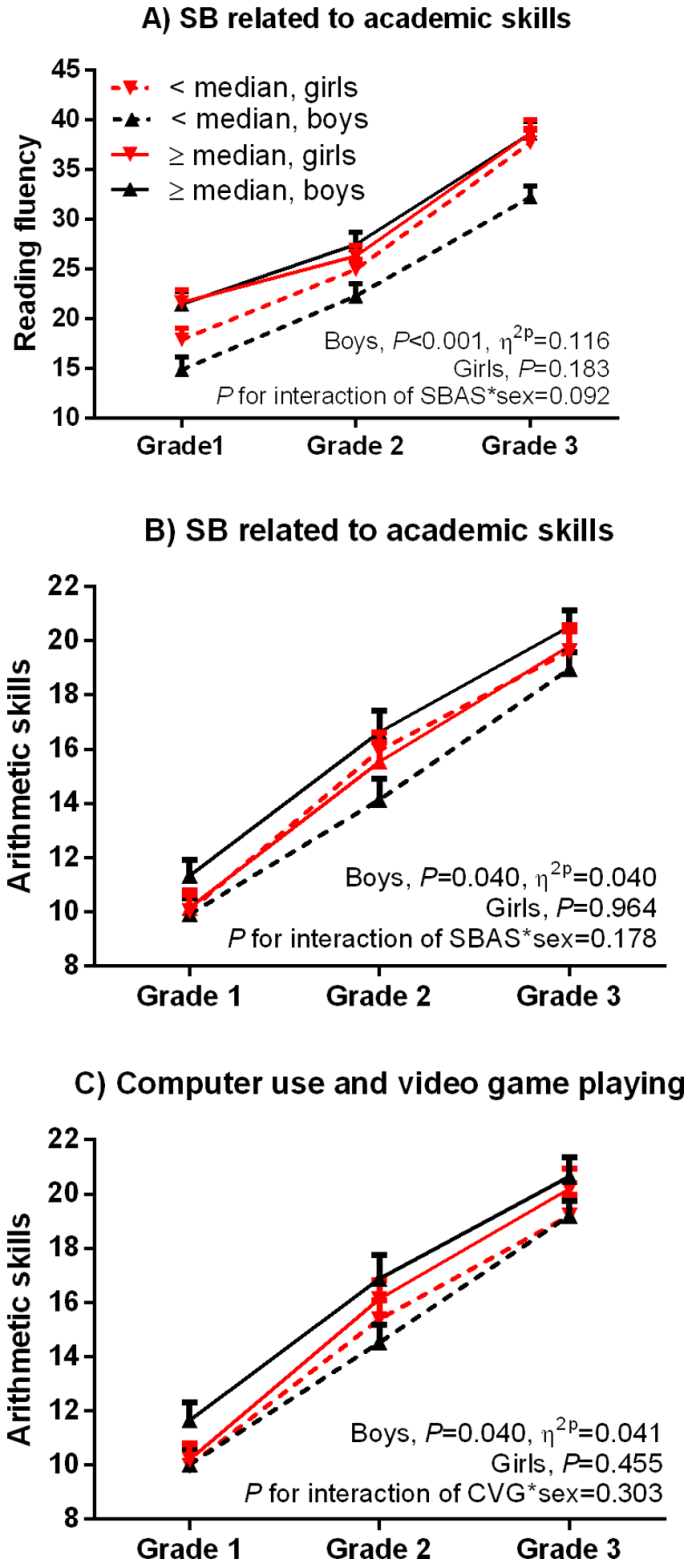
Differences in academic skills in Grades 1–3 between children who were in the upper and lower halves of sedentary behavior related to academic skills (A and B) and computer use and video game playing (C) in Grade 1 among 107 boys and 79 girls. Data are from analyses of covariance with repeated measures adjusted for age, parental education and the PANIC study group (intervention vs. control). SB = Sedentary behavior; CVG = Computer use and video game playing; SBAS = Sedentary behavior related to academic skills. Error bars represent standard errors of the mean (SEM).

Sedentary behavior related to academic skills as a continuous variable was directly associated with reading fluency in Grades 1–3 and with reading comprehension in Grade 1 after adjustment for age, parental education and the PANIC study group (Table 3). Boys who were in the upper half of sedentary behavior related to academic skills in Grade 1 (≥median of 23 min/d) had better reading fluency and arithmetic skills in Grades 1–3 than those who were in the lower half after these adjustments (Figure 2, A and B). Further adjustments had no effect on these associations or differences (data not shown).

Using computer and playing video games in Grade 1 as a continuous variable was not associated with academic skills after adjustment for age, parental education and the PANIC study group (Table 3). However, boys who were in the upper half of computer use and video game playing in Grade 1 (≥median of 39 min/d) had better arithmetic skills in Grades 1–3 than those who were in the lower half after these adjustments (Figure 2, C). These differences were no longer statistically significant after further adjustment for motor performance (data not shown). Other adjustments had no effect on these differences (data not shown).

Sitting and lying for a rest as a continuous variable was directly related to reading comprehension in Grade 1 after adjustment for age, parental education and the PANIC study group (Table 3). Further adjustment had no effect on these associations or differences (data not shown).

### Sedentary behavior and academic skills among girls

Total sedentary behavior and sedentary behavior related to music as continuous variables were inversely associated with arithmetic skills in Grades 2–3 after adjustment of age, parental education and the PANIC study group (Table 3). Also sedentary behavior related to arts, crafts and games as a continuous variable was inversely associated with arithmetic skills in Grade 3 after these adjustments (Table 3). Further adjustments had no effect on these associations.

## Discussion

This follow-up study provides new evidence for the associations of different types of PA and sedentary behavior in Grade 1 with reading and arithmetic skills in Grades 1–3 in a population sample of Finnish children. In the whole sample of children, higher levels of PA during recess and physically active school transportation were associated with a better reading fluency, any engagement in organized sports was related to better arithmetic skills and sedentary behavior related to academic skills was associated with a better reading fluency. Among boys, higher levels of total PA and physically active school transportation were associated with a better reading fluency and reading comprehension, sedentary behavior related to academic skills was related to a better reading fluency and reading comprehension and better arithmetic skills and higher levels of computer use and videogame playing were related to better arithmetic skills. Among girls, total PA and different types of PA had weak inverse or no associations with academic skills, but also total sedentary behavior and sedentary behavior related to music and arts, crafts and games were inversely associated with arithmetic skills.

Our observations are in line with increasing evidence that higher levels of PA are associated with a better academic achievement in children [9], [45]. However, our findings suggest that only some types of PA may improve academic skills among children. Higher levels of recess PA were associated with a better reading fluency in the whole sample of children. Although this is the first study to reveal the direct association of recess PA with a measure of academic achievement, the results of earlier studies suggest that recess PA enhances attention, concentration and on-task behavior which may improve academic achievement in children [28]. Recess offers not only an opportunity to exercise but also a break period from concentrated teaching in the class-room that may enhance children’s ability to re-concentrate afterwards [46]. In all Finnish schools recesses are offered throughout the school day. Therefore, higher levels of PA during recess may markedly improve reading achievement among children. Our finding is also indirectly supported by the observation that a single PA bout of 20 minutes improves reading achievement in children with and without attention deficit or hyperactivity disorder symptoms [21], [22]. Recess PA may also offer socioemotional benefits, such as improved peer relationships and an improved social climate at school, which may improve academic achievement [46]. It should also be noted that 1.5 hours of physical education per week are compulsory during Grades 1–9 in Finland. Because this amount of physical education was uniform among all children, we could not analyze the associations of PA during physical education with academic achievement.

We found that any engagement in organized sports was related to better arithmetic skills in the whole sample of children. The results of some other studies have also suggested that sport participation improves academic achievement, but some other studies have reported no such association [9], [20], [28]. There are a few biological mechanisms by which PA and sport participation could improve cognition and academic achievement. PA has been found to increase serum and plasma concentrations of brain-derived neurotrophic factor (BDNF), insulin-like growth factor-1 (IGF-1) and vascular endothelial growth factor (VEGF) and may thereby improve hippocampal neurogenesis, synaptic plasticity, synaptic transmission, cerebral blood flow and neural survival [47]–[49]. Participation in organized sports may also improve self-regulatory skills, such as self-monitoring, evaluation and effort, and could thereby promote academic achievement [50].

Total PA was directly related to reading fluency and reading comprehension across the first three grades of primary school among boys. However, the associations of total PA with reading skills were partly explained by body adiposity and physical performance that have earlier been related to academic achievement and cognition in children [40], [51], [52]. Our findings are consistent with the results of a recent study in which total PA at the age of 11 years was directly related to academic achievement at the ages of 13 and 16 [53].

We observed that boys with higher levels of physically active school transportation had a better reading fluency and reading comprehension than other boys. These differences remained even after further controlling for most other determinants of academic skills. However, these relationships became less evident when motor performance was taken into account. Motor performance has earlier been shown to be a strong determinant of academic achievement [40], [54]. To our knowledge, there are no previous studies on the associations of physically active school transportation with measures of academic achievement. However, one study reported that adolescents who spent more time in commuting to and from school by walking or bicycling had a better cognitive performance than those who spent less time in physically active commuting [29]. Some other studies have provided evidence that short bouts of PA transiently improve cognitive functions, such as attention and concentration, and neuroelectric processing among children and adolescents [24], [55]. Moreover, acute exercise has been observed to increase plasma concentrations of BDNF [49]. Another study showed acutely reduced cardiovascular stress responses and reduced perceived stress during a cognitive task among children who walked at moderate intensity before the task but not among children who were sedentary before the task [56]. Repeated bouts of PA, such as physically active school transportation, have also been found to improve brain function and cognition especially when accompanied with cognitive challenges such as school lessons [57]. Thus, boys who commute to school by walking or bicycling may concentrate better and feel less stress during a school day and thereby have a better academic achievement than boys who do not commute.

We found only a few and weak inverse associations of different types of PA with academic skills among girls, and these relationships were largely explained by body adiposity. However, there were inverse associations of total PA with academic skills among girls whose parent had no academic degree, whereas total PA was directly related to academic skills among girls whose parents had an academic degree. There is also some previous evidence that a higher parental education is linked to higher levels of PA [58] and better early reading skills [59] in children. Parental encouragement and support to be physically active, such as the transportation of children to physically active hobbies, have been associated with higher levels of PA in girls from families with a higher socioeconomic status, but not in boys or girls from families with a lower socioeconomic status [60]. It is possible that engagement in PA is more desired in more educated families than in families with less educated parents. Higher educated parents may also feel more social pressure to encourage their children to participate in PA than lower educated parents. In higher educated families, physically more active girls may also receive more educational support from their parents than physically less active girls. However, less educated parents may encourage their children to engage in sedentary activities that support educational ability rather than PA. One explanation for the modification of the associations of PA with academic skills by parent’s education may be differences in the support of the children’s psychosocial development, such as body image and self-esteem, between families [9].

Our results regarding the associations of different types of sedentary behavior with academic skills are partly consistent with the existing evidence [30]. However, we found no association between watching TV or videos and academic achievement, which is in contrast to the results of previous studies [30]. Tremblay and coworkers [30] suggested that over two hours of screen time per day is associated with a worse academic achievement. One reason for not observing the association between watching TV or videos and academic achievement in our study may be that a relatively small proportion of children in our study sample spent this much time watching TV or videos. Furthermore, there is some evidence that screen time is more strongly related to academic achievement in adolescents than in children [61].

Using computer and playing video games were related to better arithmetic skills in boys. One explanation for this observations is that computer use and video game playing were more common in boys than in girls and that these activities include counting, recognizing numbers and tasks requiring visuospatial perception which improve arithmetic skills in children [62]. Our finding is in line with the results of earlier studies suggesting that computer use may be beneficial for academic skills among children [31], [63]. However, the association of computer use and video game playing with arithmetic skills in boys was partly explained by motor performance that was directly related to academic skills in the present study sample of children [40].

We found that more time spent in sedentary behavior related to academic skills, which included reading and writing, was related to a better reading and arithmetic skills in boys. This observation is consistent with previous evidence that children who spent more time in reading with their mothers in preschool age had better reading skills than other children [64]. On the other hand, we found that girls who spent more time in sedentary behavior related to music and sedentary behavior related to arts, crafts and games had worse arithmetic skills than other girls. Inconsistent with our findings, the results of another study suggested that a long-term engagement in a musical hobby improves academic skills and cognition among children [65]. Our results suggest that the associations of different types of sedentary behavior with academic skills are complicated and may differ between sexes. Therefore, studies in large population samples of girls and boys are needed to clarify differences in the relationships of different types of sedentary behavior with academic skills between sexes as well as the interactions of sedentary behaviors with other determinants of academic achievement, such as body adiposity, motor performance and psychosocial factors.

The strengths of the present study include a follow-up during the first three school years and the comprehensive assessment of different types of PA and sedentary behavior and the assessment of academic skills using standardized tests. However, although we had a prospective study design, our study cannot prove causal relationships of PA or sedentary behavior with academic skills. It is possible that children’s PA and other behaviors had changed during the follow-up period and had influenced the observed associations. Moreover, we did not assess the content of TV programs watched by the children, and did not separate playing physically active and passive videogames, which may have influenced the associations of sedentary behavior with academic skills. A relatively small population sample of primary school children weakens the generalizability of our observations and the relatively large number of regression models used increases the likelihood of false positive findings, particularly in analyses stratified by sex. Another limitation of our study is that we did not measure PA and sedentary behavior with accelerometers or other objective methods. However, total PA measured by the PANIC Physical Activity Questionnaire had a moderate positive correlation with total PA measured objectively by the Actiheart monitor in a subset of the children, suggesting that our questionnaire can be used for the assessment of total physical activity among children. Moreover, we designed the questionnaire specifically for investigating the different types of PA and SB among primary school children. In fact, questionnaires are currently the only feasible method for assessing the different types of PA and sedentary behavior in population samples [66]. The PANIC Physical Activity Questionnaire was specifically designed for investigating the different types of PA and sedentary behavior among children. There is some evidence that vigorous PA is needed to improve academic achievement particularly in female adolescents [7], and that may partly explain the few inconsistent associations of PA with academic skills among girls in our study.

## Conclusions

More time spent in PA during recess, physically active school transportation, engagement in any organized sports and sedentary behavior related to academic skills were associated with better academic skills during the first school years in children. Higher levels of total PA, physically active school transportation, computer use and video game playing and sedentary behavior related to academic skills were related to better academic skills during the first school years in boys. However, higher levels of total sedentary behavior, sedentary behavior related to music and sedentary behavior related to arts, crafts and games but also higher levels of total PA were associated with worse academic skills in girls. These findings suggest that there are differences in the benefits of PA and other hobbies for the development of academic skills during the first school years among boys and girls. Larger prospective epidemiological studies and intervention studies are warranted to provide further evidence for the possible causal associations of different types of PA and sedentary behavior with academic achievement in boys and girls.
